# Surgical management of intramuscular hemangioma of the foot: a case report

**DOI:** 10.1186/s13037-019-0197-1

**Published:** 2019-03-26

**Authors:** K. Lahrach, S. Abdulrazak, A. Marzouki, F. Boutayeb

**Affiliations:** Department of Trauma and Orthopedic Surgery A, Hassan II Teaching Hospital, Faculty of Medicine and Pharmacy, Sidi Mohammed Ben Abdellah University, Atlas, Avenue Hassan II, 1835 Fès, BP 30000 Morocco

**Keywords:** Intramuscular, Hemangioma, Plantar, Excision, Sclerotherapy

## Abstract

**Background:**

Hemangiomas are benign tumors usually found in the lower extremity yet their surgical management on the location in the foot is rarely documented.

**Case presentation:**

We report a case of a plantar intramuscular hemangioma in 25-year-old patient with a history of percutaneous therapy. Patient had undergone intralesional sclerotherapy 3 years prior to his admission with persistent pain on weight bearing activities.

MRI demonstrated a multi lobulated lesion of the 1st IMS with a peripheral enhancement on gadolinium injection. The patient underwent elective surgery with complete excision and no functional impairment at the last follow-up 3 years after surgery.

**Conclusion:**

Intramuscular hemangiomas are rare occurrences. Steroid injection and sclerotherapy are effective non-operative methods. Complete excision of isolated hemangioma lesions allows definite diagnosis with no recurrence.

## Background

Intramuscular hemangiomas (IMH) are benign neoplasms accounting for less than 1% of all hemangiomas [1] All muscles could be affected yet those of thigh are the most involved [2]. Foot localizations are poorly documented with a few cases reported in literature [3–5]. Its etiopathogenesis and variable presentation could make it a source of diagnostic dilemma [1, 6]. Steroid injections and intralesional sclerotherapy are effective non-operative methods. Operative management of symptomatic plantar IMH is compounded by tumor size at diagnosis and its close links with the neurovascular bundles and tendons of the foot. The authors highlight the challenges and pit falls in the management of IMH through a case report with reference to relevant literature.

## Case presentation

The patient is a 24-year-old male with no prior trauma who presented with a 7 year history of forefoot pain placed on several over-the-counter drugs and other conservative treatments including percutaneous therapy. The patient recalled steroid injection and intralesional sclerotherapy three years prior to her admission with no relief. Physical examination demonstrated a firm mass on the plantar surface of the first intermetatarsal space (IMS) without motor nor sensory deficit. Vascular examination was unremarkable.

**Fig. 1:**
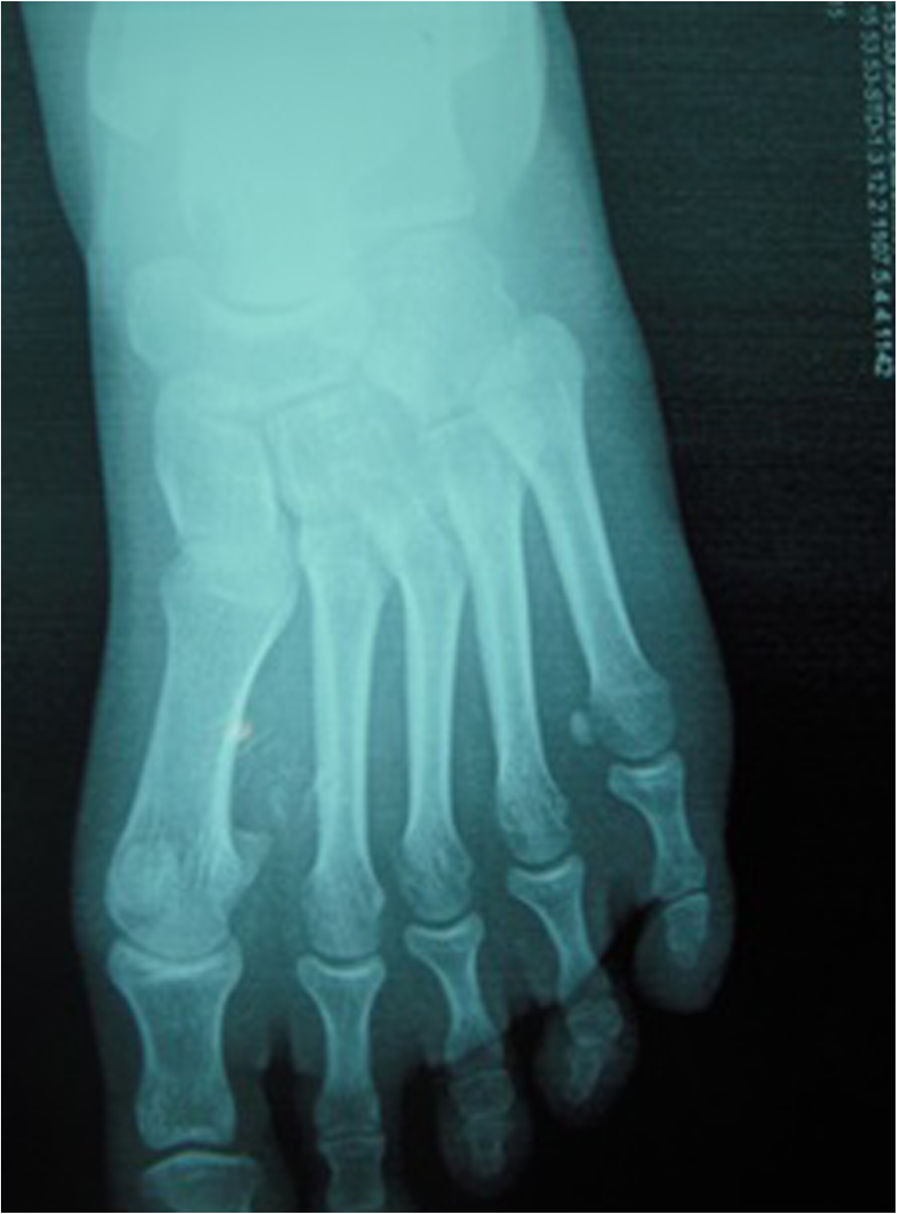
Plain X-ray showing a widening of the 1st IMS with phleboliths

**Fig. 2:**
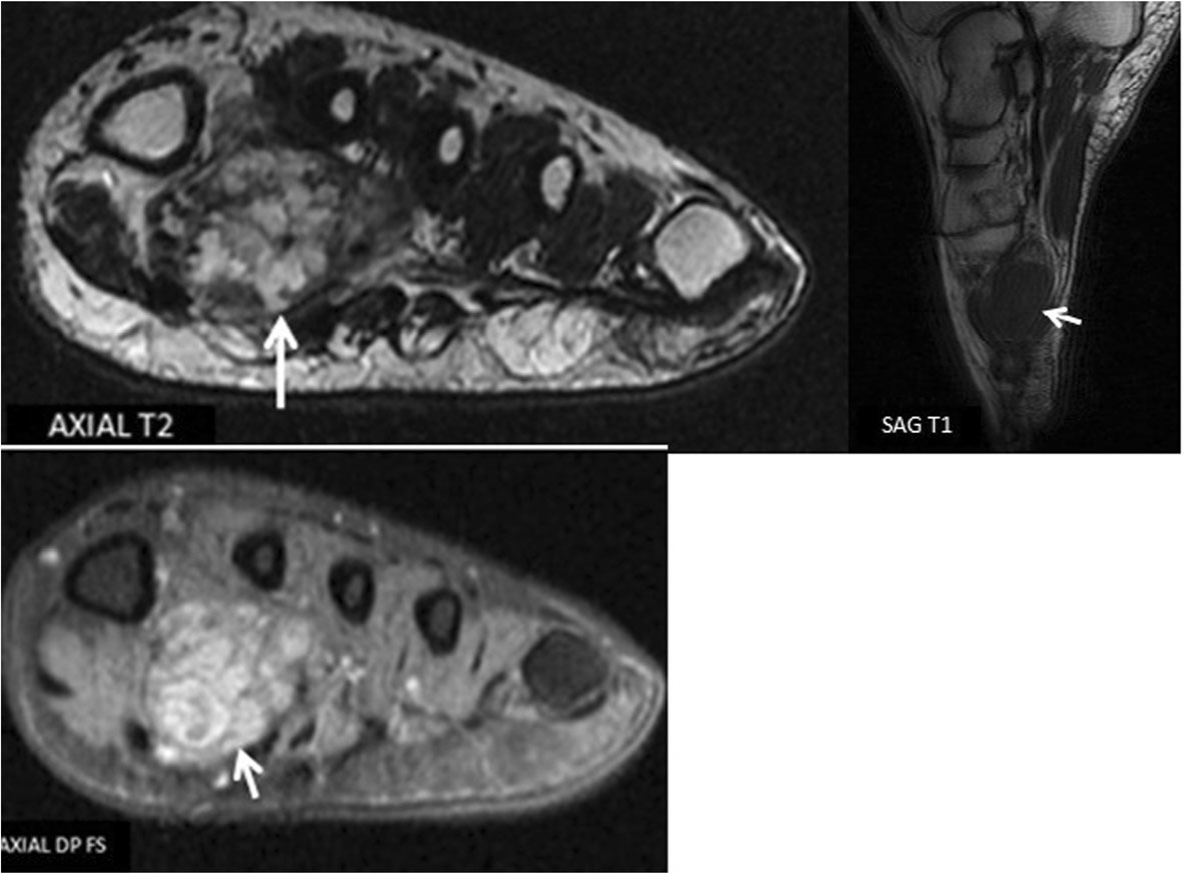
Foot MRI showing a multi-lobulated hemangioma on the plantar side of the foot taking up the first two metatarsals

Plain x-ray demonstrated a soft tissue widening of the 1st IMS with phleboliths (Fig. 1). The surrounding bony structures of the forefoot were unaffected. Magnetic Resonance Imaging (MRI) demonstrated a hypo intense lesion on T1 weighted sequence taking up the plantar surface of the left foot.. There was peripheral heterogeneous enhancement after gadolinium injection. The lesion was multi-lobulated, measuring 30 × 22 mm, with intermediate intensity and contained several separations on T2 weighted and fat suppression sequences. The lesion had completely taken up the 1st IMS displacing the soft tissues of the midfoot without any signs of infiltration (Fig. 2).

**Fig. 3:**
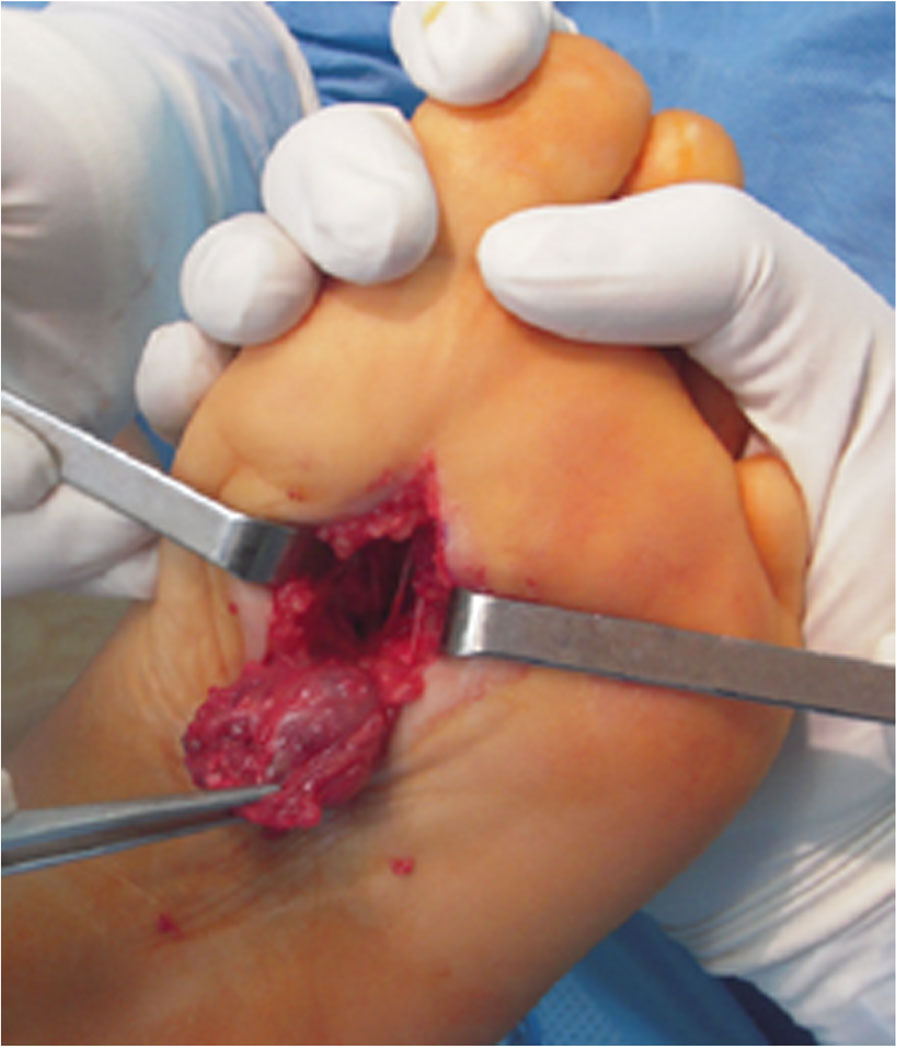
peroperative image showing complete excision of highly vascularized tumour

The patient underwent surgery after written and informed consent was obtained. A plantar longitudinal approach across the 1st interosseous metatarsal space was undertaken. After careful dissection, an intramuscular tumor taking up the interosseous muscles was excised. (Fig. 3). Immediate postoperative recovery was uneventful. Pathology examination of the surgical specimen demonstrated an intramuscular hemangioma without any signs of malignancy. Patient has no signs of local recurrence or functional impairment of the foot at the last follow-up, 3 years after complete excision.

## Discussion

Intramuscular hemangioma or angioma (IMH) is a benign vascular neoplasm of the skeletal muscle. It occurs in young adults before the third decade with a slight feminine predominance observed in most cases [2] .Malignant transformations remain exceptional and metastases have never been previously reported [7]. In adults, its prevalence is believed to be very low, representing less than 1% of soft tissue tumors [8]. Weiss and Enzinger [9] reported only ten intramuscular forms on 570 hemangioma specimens.

Although its pathogenesis still remains unclear, a congenital origin is often suggested [10] and a history of trauma is usually present in most reported cases. IMH can develop in all skeletal muscles, yet those of the thigh are the most commonly affected. Wild et al. [11] found that the quadriceps muscle represents the most common involvement presenting as a painful mass or incipient swelling of the lower limb.

A palpable mass is found on physical examination at time of diagnosis in 98% of reported cases [8]. The mass may present with classic signs of a vascular tumor, notably bluish discoloration of the skin with increased local temperature or superficial dilated veins presenting as mass discolored on compression. Large hemangiomas may be accompanied with thrills and bruit. IMH is usually longstanding and slow growing over a long period of time. In the course of its development certain complications may arise including bleeding, hematoma, thrombosis and nerve compression sometimes responsible for misdiagnosis.

Plantar intramuscular hemangiomas are seldom reported in literature. Wisniewski et al. [12] reported a case of IMH in an adult athlete whereas Wild et al. [11] related a case of cavernous hemangioma involving flexor digitorum brevis of the foot.

Plain films may reveal phleboliths that are round lumps of calcifications primarily due to thrombosis within the lesion [13]. MRI is currently the gold standard of imaging, with preoperative findings frequently diagnostic helps to define the location and links of the tumor [14]. Seven histological subtypes can be identified depending on their composition-cavernous, capillary, venous, arteriovenous, epitheloid, granulation tissue type and miscellaneous. They may be either localized or diffuse (angiomatosis) [2].

Surgical excision is the mainstay treatment [7] as it also allows definite diagnosis. Complete surgical excision with surrounding margin of normal muscle is considered optimal treatment and reduces the risk of recurrence. Recurrence rates vary from 18 to 61% [15] with tumor size and resection margins considered major risk factors. Large hemangiomas may not be amenable to complete excision.

Compressive sclerotherapy, using a variety of products, radiotherapy and laser ablation have also been suggested as treatment options in large diffuse hemangiomas where excision is impractical. Uslu et al. [16] demonstrated the usefulness of percutaneous sclerotherapy as conservative treatment of symptomatic plantar IMH with less morbidity and how this treatment could be repeated if necessary. Embolization may be also be indicated as an adjunct to surgery to reduce the risk of intraoperative hemorrhage of large hemangiomas [17]. In cases of infiltrating hemangioma where complete excision cannot be undertaken due to extensive invasion, embolization could prove beneficial and welcome addition to popular non-conservative treatments.

## Conclusion

IMH are rare benign tumors that do not undergo spontaneous regression. Plantar IMH are nuisance to weight bearing activities and their management could cause a therapeutic dilemma. This case report demonstrates a unique presentation of intramuscular hemangioma due to its localization. The hemangioma was surgically excised with no recurrence or functional impairment of the foot.

## Abbreviations

IMH: Intramuscular Hemangioma
IMS: intermetatarsal space
MRI: Magnetic Resonance Imaging
